# Effects of biogenic silver and iron nanoparticles on soybean seedlings (*Glycine max*)

**DOI:** 10.1186/s12870-022-03638-1

**Published:** 2022-05-24

**Authors:** Mariana Guilger-Casagrande, Natália Bilesky-José, Bruno Teixeira Sousa, Halley Caixeta Oliveira, Leonardo Fernandes Fraceto, Renata Lima

**Affiliations:** 1 Laboratory for Evaluation of the Bioactivity and Toxicology of Nanomaterials, University of Sorocaba (UNISO), Rod. Raposo Tavares, km 92.5 – Vila Artura, Sorocaba, São Paulo, 18023-000 Brazil; 2 Laboratory of Environmental Nanotechnology, São Paulo State University (UNESP), Av. Três de Março 511, Sorocaba, São Paulo, 18087-180 Brazil; 3 Departament of Animal and Plant Biology, University of Londrina (UEL), Rod. Celso Garcia Cid km 380, Londrina, Paraná, 86057-970 Brazil

**Keywords:** Biogenic metallic nanoparticles, AgNPs, FeNPs, Soybean, Phytotoxicity, Lignification

## Abstract

**Background:**

Biogenic metallic nanoparticles have been emerging as a promising alternative for the control of phytopathogens and as nanofertilizers. In this way, it is essential to investigate the possible impacts of these new nanomaterials on plants. In this study, the effects of soil contamination with biogenic silver (AgNPs) and iron (FeNPs) with known antifungal potential were investigated on morphological, physiological and biochemical parameters of soybean seedlings.

**Results:**

The exposure of plants/seedlings to AgNPs induced the reduction of root dry weight followed by oxidative stress in this organ, however, adaptive responses such as a decrease in stomatal conductance without impacts on photosynthesis and an increase in intrinsic water use efficiency were also observed. The seedlings exposed to FeNPs had shown an increase in the levels of oxygen peroxide in the leaves not accompanied by lipid peroxidation, and an increase in the expression of *POD2* and *POD7* genes, indicating a defense mechanism by root lignification.

**Conclusion:**

Our results demonstrated that different metal biogenic nanoparticles cause different effects on soybean seedlings and these findings highlight the importance of investigating possible phytotoxic effects of these nanomaterials for the control of phytopathogens or as nanofertilizers.

## Background

The use of metallic nanoparticles in agriculture, both for the control of pathogenic microorganisms and the stimulation of plant growth has been standing out as a promising tool that has a greater health and environmental safety as a priority [1–4]. Metallic nanoparticles are considered less impacting in comparison with conventional agrochemicals due to their efficacy in low concentrations and the fact that successive applications are not necessary [5, 6]. However, despite the lower impact, these nanoparticles are commonly synthesized through chemical methods which employ toxic substances, triggering residual toxicity [6].

Biogenic metallic nanoparticles are synthesized by the products of the metabolism of bacteria, fungi and plants, which makes them an environmentally friendly alternative that shows lower impacts on human health and non-target organisms [7, 8]. These nanoparticles possess a capping of biomolecules that confers them higher stability, biocompatibility and lower toxicity [9], making them a promising alternative both for the control of phytopathogens and the stimulation of the development of agricultural crops [10–12]. However, despite this potential, few studies investigate the possible phytotoxic effects of biogenic metallic nanoparticles on the most important agricultural crops.

The effects of metallic nanoparticles on plants are determined by several factors such as the metal type, size, morphology, exposure concentration, capping composition and aggregation state [13]. In the case of biogenic nanoparticles these parameters may vary substantially according to the reducing and stabilizing agent employed in the synthesis, giving them unique characteristics and properties [7, 14, 15]. In addition, the effects may vary according to the plant species and the stage of development, the different exposed tissues and the kind of exposure [16–18].

Some studies report low phytotoxic effects of biogenic metallic nanoparticles on different plant species [19, 20]. Anwar et al. (2021) observed a reduction in germination index, root and shoot length, content of photosynthetic pigments and total proteins of *Vigna radiata* seedlings exposed to biogenic silver nanoparticles [19]. Verma et al. (2020) observed toxic effects of biogenic silver nanoparticles on *Phaseolus vulgaris* when the seeds were exposed to the highest concentrations of the nanoparticles [20]. In contrast, the majority of the studies show the positive effects of these new nanomaterials on plants such as antioxidant activity and growth promotion [21–25]. Noshad et al. (2019) observed an increase in germination rate and growth parameters of *Solanum lycopersicum* treated with silver nanoparticles synthesized using the filtrates of the fungi *Trichoderma harzianum* and *Aspergillus fumigatus* [21]. Kannaujia et al. (2019) observed the antioxidant potential and growth promoting effect of biogenic silver nanoparticles on wheat plants [22]. Tovar et al. (2020) evaluated the effects of biogenic iron nanoparticles on corn germination and found positive results such as a higher germination index and an increase in root and shoot length [23]. Win et al. (2021) observed an increase in the germination index of rice, corn, mustard, green gram, and watermelon seeds treated with biogenic iron nanoparticles [24].

In previous studies by our group the synthesis of biogenic silver nanoparticles (AgNPs) and iron nanoparticles (FeNPs) was performed employing the biocontrol fungus *Trichoderma harzianum* as a reducing and stabilizing agent. Both the nanoparticles showed high potential for the control of mycelial growth and the formation of sclerotia of the phytopathogenic fungus *Sclerotinia sclerotiorum *in vitro, with low toxicity on cell lines and non-target organisms [26, 27]. Given this promising effect of the nanoparticles against the phytopathogen, it is worth investigating the possible impacts of these nanoparticles on the development and physiology of plants, ensuring safe application. The aim of the present study was to evaluate the effects of AgNPs and FeNPs synthesized in our previous studies on morphological, physiological and biochemical parameters of soybean seedlings.

## Material and methods

### Biogenic metallic nanoparticles

The silver (AgNPs) and iron (FeNPs) nanoparticles employed in the present study were synthesized in our previous studies using the filtrate of the fungus *Trichoderma harzianum* as reducing and stabilizing agent. Briefly, the synthesis consisted in the initial culture of the microorganism in agar medium followed by the culture in broth medium, harvesting and transference of the biomass into water, and collection of the filtrate. Silver nitrate (AgNO_3_) and iron (III) chloride (FeCl_3_) were added to the filtrate as metallic precursors to the final concentration of 1 mM, giving rise to AgNPs and FeNPs, respectively.

The characteristics by dynamic light scattering (DLS) and microelectrophoresis are mean hydrodynamic diameter 81.84 ± 0.67 nm, polydispersity index (PDI) 0.52 ± 0.00 and zeta potential -18.30 ± 1.73 mV, for AgNPs, and mean hydrodynamic diameter 207.30 ± 2.0 nm, PDI 0.45 ± 0.07 and zeta potential 13.4 ± 2.0 mV, for FeNPs.

### Soil exposure to the nanoparticles, soybean sowing and cultivation

The effects of the biogenic metallic nanoparticles on soybean (*Glycine max* L. Merr. Cv. BRS 257) were evaluated in the State University of Londrina (UEL), Department of Animal and Plant Biology, Paraná, Brazil. This plant species was chosen due to its worldwide economic importance as a source of food and protein, the easy cultivation and the fact that this crop is commonly affected by phytopathogens, which may be controlled by nanomaterials such as metallic nanoparticles.

The characteristics of the soil used in the experiment were as follows: pH in CaCl_2_—5.8, organic matter—4 g dm^−3^, P—7 mg dm^−3^, K—0.04 cmolc dm ^−3^, Ca—0.8 cmolc dm^−3^, Mg—0.7 cmolc dm^−3^, H + Al_2_ 2 cmolc dm^−3^, SB (sum of bases)—1.5 cmolc dm^−3^, CTC—3.5 cmolc dm^−3^, V% (base saturation)—44.

Initially, the soil was exposed to the AgNPs and FeNPs, separately, in plastic pots (14 cm in upper diameter, 9.5 cm in lower diameter and 10.5 cm in height). The treatment was carried out by pouring the suspensions of nanoparticles on soil surface and mixing to a depth of 5 cm, then the soybean seeds were sown at a depth of 3 cm. The quantity of nanoparticles was 1.53 × 10^13^ NPs/m^2^ for AgNPs and 2.35 × 10^11^ NPs/m^2^ for FeNPs, based on the effective quantity for the control of *Sclerotinia sclerotiorum in vitro* [26, 27]. Five pots were prepared, with five seeds per pot. As a control, the same number of pots were prepared, with the same number of seeds per pot, in soil free of nanoparticles.

The pots were kept in greenhouse in a randomized design at natural light conditions for 25 days (from April 25^th^ 2019 to May 20^th^ 2019), with daily watering. The average monthly values of temperature and accumulated global solar radiation during the experiment were 21.5 ± 1.2 °C and 358.5 ± 25.5 MJ m^−2^, respectively [28]. Twelve days after sowing, the soil was supplemented with 50 mL based on Hoagland and Arnon’s (1950) nutrient solution (1 mM KH_2_PO_4_, 4 mM Ca(NO_3_)2.4H_2_O. 2 mM K_2_SO_4_, 4 mM (NH_4_)_2_SO_4_, 2 mM MgSO_4_.7H_2_O, 92.5 µM H_3_BO_3_, 18 µM MnCl_2_.4H_2_O, 1.5 µM ZnCl_2_, 0.56 µM Na_2_MoO_4_.2H_2_O, 0,66 µM CuCl_2_.2H_2_O, 100 µM FeSO_4_) [29].

### Leaf gas exchange

Leaf gas exchange parameters were recorded 25 days after sowing, on a sunny day, between 09:00 and 11:00 am, using an infrared gas analyzer (IRGA) system (LI-6400XT, LI-COR Biosciences, Lincoln, NE, USA) connected to a 6 cm^2^ 6400-02B measuring chamber with LED light source where the leaves were exposed to a saturating photosynthetically active radiation (PAR) of 1500 μmol m^−2^ s^−1^. The central leaflet of the youngest fully expanded leaf of two randomly selected seedlings from each pot was chosen. From this analysis, the rates of net photosynthesis and stomatal conductance were obtained, and the ratio between these measurements was calculated to obtain the intrinsic water-use efficiency.

### Photosynthetic leaf pigments

For the analysis of photosynthetic pigments, 0.05 g of freshly collected leaves were ground to a powder in liquid nitrogen and 5 mL of acetone solution (80%) in sodium phosphate buffer (2.5 mM; pH 7.8) were added, maintaining the samples on ice. Then, vortexing was performed followed by centrifugation at 1800 xg for 10 min. The absorbance of the supernatant was measured at 663.2 nm, 646.8 nm and 470 nm to determine the levels of chlorophyll a, chlorophyll b and carotenoids, respectively, using the Eqs. 1, 2 and 3, proposed by Wellburn (1994), with constant values for acetone extraction [30].

1 $$Ca=12.25\times A663.2-2.79\times A646.8$$

2 $$Cb=21.5\times A646.8-5.1\times A663.2$$

3 $$Cx+c=\frac{(1000\times A470-1.82\times Ca-85.02\times Cb)}{198}$$

where *Ca* = Chlrophyll a, *Cb* = Chlrophyll b, *Cx* + *c* = Carotenoids, and A663,2, A646,8 and A470 nm are the absorbances obtained in the respective wavelengths.

### Morphological analysis

For morphological analysis, two seedlings from each pot were randomically selected and measurements were made of the shoot length, root length, and leaf area (LI3000C leaf area meter, LI-COR © Biosciences, Lincoln, USA). For weight analysis, shoots and roots were harvested and kept for 72 h at 60 °C, prior to dry weight measurement.

### Biochemical analysis

Hydrogen peroxide (H_2_O_2_), malondialdehyde (MDA) and conjugated dienes were measured in leaves and roots as markers of oxidative stress. For the extraction of H_2_O_2_ and MDA, the plant tissues (0.1 g) were ground to a powder in liquid nitrogen and homogenized with 1 mL of trichloroacetic acid (0.2%) diluted in cold methanol. After centrifugation at 13,700 xg, at 4ºC for 5 min, the supernatant was used to measure H_2_O_2_ through the reaction with 1 M potassium iodide in phosphate buffer [31] and MDA through the determination of thiobarbituric acid (TBARS) reactive substances [32]. For extraction of conjugated dienes, the plant tissues (0.1 g) were ground to a powder in liquid nitrogen and homogenized with 1 mL of cold 96% ethanol. After centrifugation at 13,700 xg, at 4ºC for 20 min, the absorbance of the supernatant was determined at 234 and 500 nm and the content of conjugated dienes estimated as described by Boveris et al. (1980) [33].

### Expression of lignification-related genes

For the analysis of the expression of lignification-related genes in the roots of soybean plants total RNA was extracted according to Bittencourt et al. (2011) [34] and the quantification was performed with Qubit™ RNA HS Assay Kit. Then, reverse transcription was performed for cDNA synthesis using SUPERSCRIPT™ III RT and the expression of the genes Phenylalanine ammonia-lyase (*PAL*), Cinnamate 4-hydroxylase (*C4H*), Cinnamyl alcohol dehydrogenase (*CAD*), Peroxidase 2 (*POD2*), Peroxidase 4 (*POD4*) and Peroxidase 7 (*POD7*) was evaluated through real-time PCR employing the ΔΔCT (2^−ΔΔCT^) method with specific primers (StepOne thermocycler) [35]. The β-actin gene was the endogenous normalizer of the analysis, as it has a constitutive expression.

### Statistical analysis

The data of morphological, physiological and biochemical parameters were compared by t test (*p* < 0.05). The data of the expression of lignification-related genes were provided with statistical analysis by the StepOne thermocycler software.

## Results

### Leaf gas exchange

**Fig. 1:**
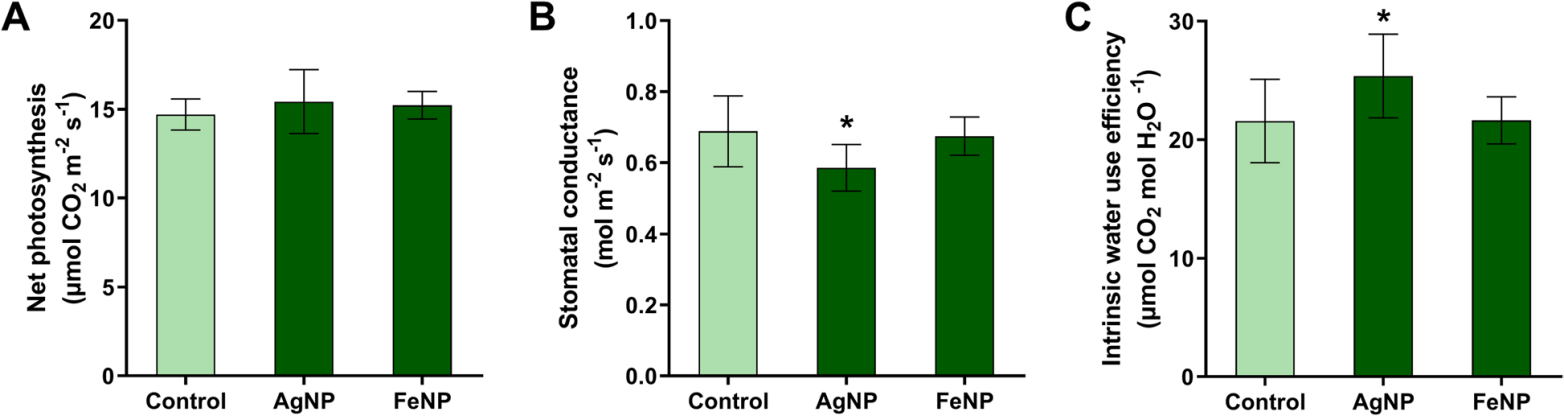
Effects of AgNPs and FeNPs on leaf gas exchange parameters of soybean seedlings. **A** Net photosynthesis; **B** Stomatal conductance; **C** Intrinsic water use efficiency. The bars are means ± standard deviation (*n* = 10). * indicates significant difference between seedlings exposed to the nanoparticles and control seedlings (*p* < 0.05)

Regarding the effects on gas exchange parameters, the nanoparticles did not cause any change in the rate of net photosynthesis. However, the exposure to the AgNPs resulted in a decrease of 15% in stomatal conductance and an increase of 19% in intrinsic water-use efficiency. FeNPs did not cause any effect on gas exchange parameters (Figs. 1A,B and C).

### Photosynthetic leaf pigments

**Fig. 2:**
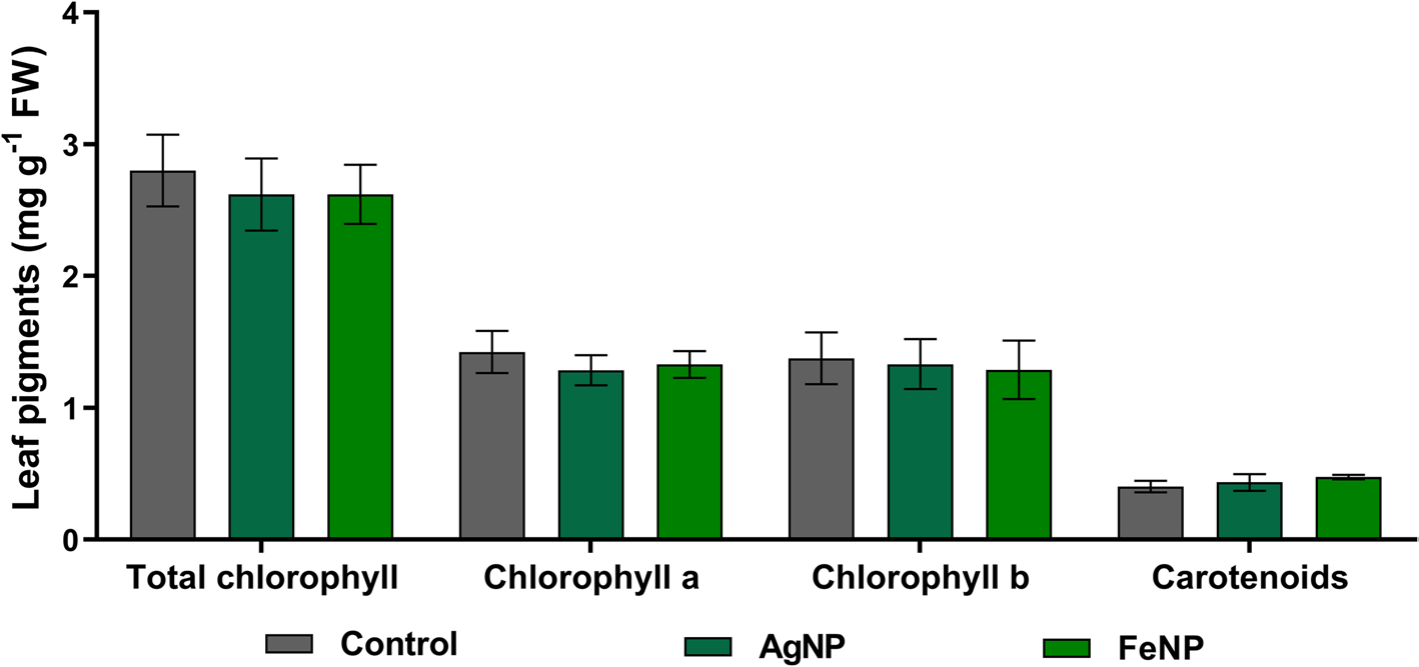
Photosynthetic leaf pigments of soybean seedlings grown in soil exposed to AgNPs and FeNPs. The bars are means ± standard deviation (*n* = 5)

The results of quantification of the photosynthetic leaf pigments showed no significant changes in the levels of total chlorophyll, chlorophyll a, chlorophyll b and carotenoids of soybean seedlings exposed to the nanoparticles (Fig. 2).

### Morphological analysis

**Fig. 3:**
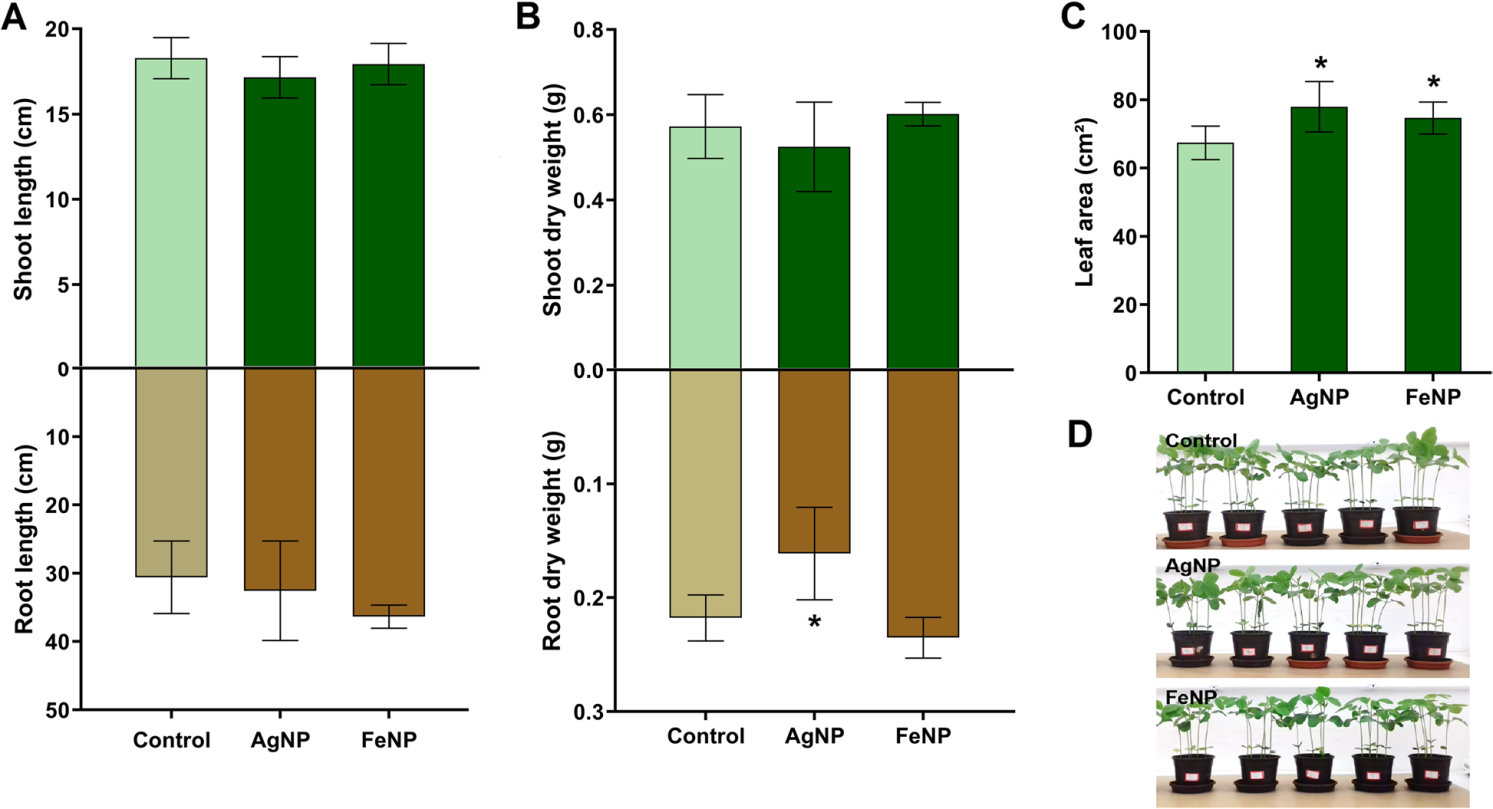
Morphological parameters of soybean seedlings cultivated in the soil exposed to the metallic nanoparticles AgNPs and FeNPs. **A** shoot and root length; **B** shoot and root dry weight; **C** leaf area; **D** aspect of the seedlings previously to collection. The bars are means ± standard deviation (*n* = 10 for length of shoot and root; *n* = 5 for shoot and root dry weight). *indicates significant difference between seedlings exposed to the nanoparticles and control seedlings (*p* < 0.05)

No changes were observed in the length of shoot and root of soybean seedlings cultivated in the soil exposed to the biogenic nanoparticles AgNPs and FeNPs (Fig. 3A), however there was a decrease of 26% in the dry weight of the roots exposed to the AgNPs compared to the control (Fig. 3B). An increase of 16% and 11% was observed in leaf area of the seedlings exposed to AgNPs and FeNPs, respectively (Fig. 3C).

### Biochemical analysis

**Fig. 4:**
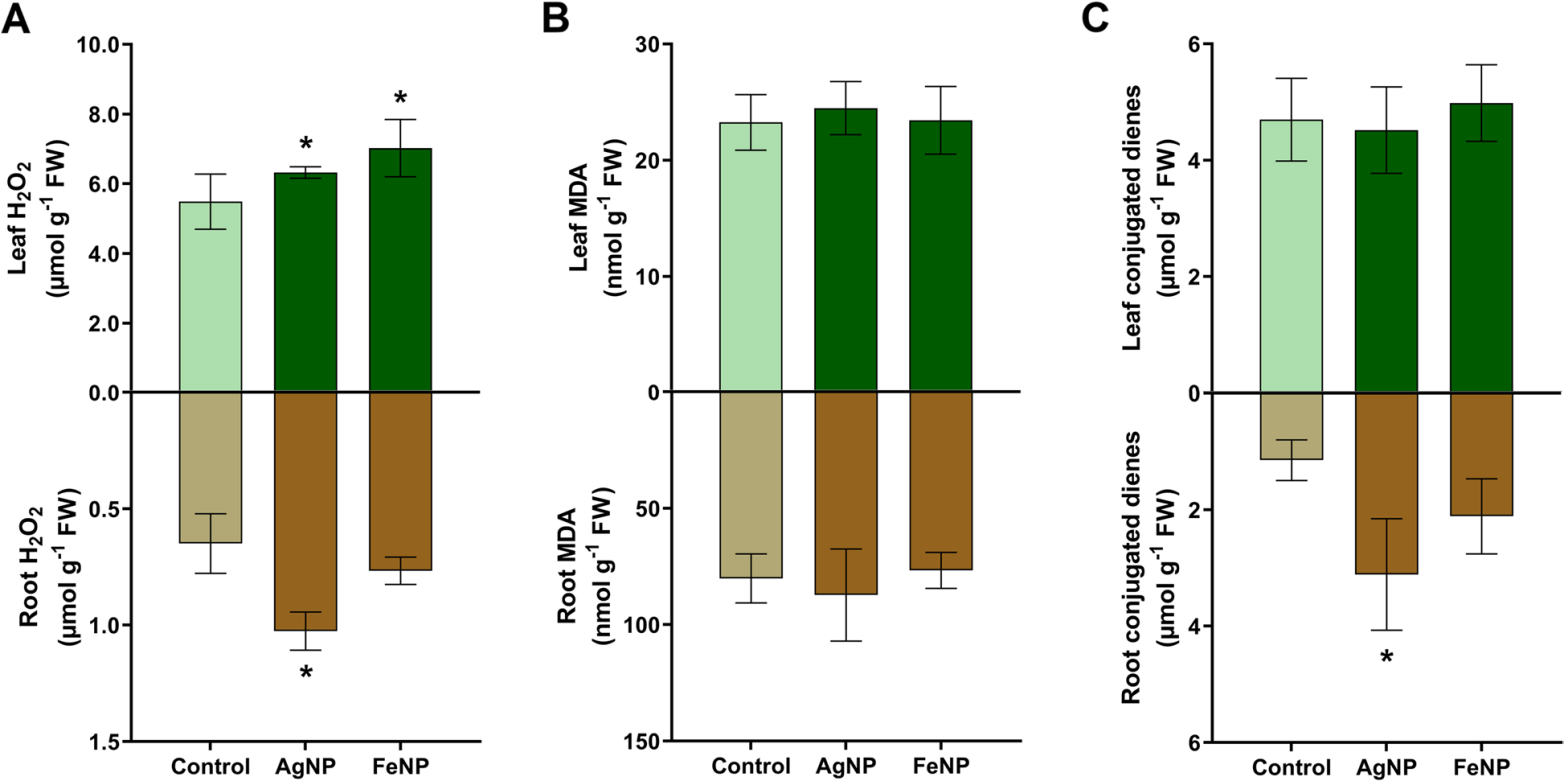
Biochemical parameters of soybean seedlings cultivated in the soil exposed to the metallic nanoparticles AgNPs and FeNPs. **A** Hydrogen peroxide (H_2_O_2_); **B** malondialdehyde (MDA); **C** Conjugated dienes. The bars are means ± standard deviation (*n* = 5). * indicates significant difference between seedlings exposed to the nanoparticles and control seedlings (*p* < 0.05)

The results of biochemical assays showed an increase of the levels of H_2_O_2_ in the leaves of the seedlings exposed to AgNPs (15%) and FeNPs (28%) compared to the control. However, the nanoparticles did not change the levels of MDA and conjugated dienes in this part of soybean seedlings, indicating absence of lipid peroxidation and major oxidative damage. In the case of the roots, the seedlings exposed to the AgNPs showed an increase in the levels of H_2_O_2_ (58%) and conjugated dienes (172%) in this organ, indicating that the formation of reactive oxygen species induced oxidative stress. No changes were observed in the levels of biochemical markers in the roots of the seedlings exposed to the FeNPs (Fig. 4).

### Expression of lignification-related genes

**Fig. 5:**
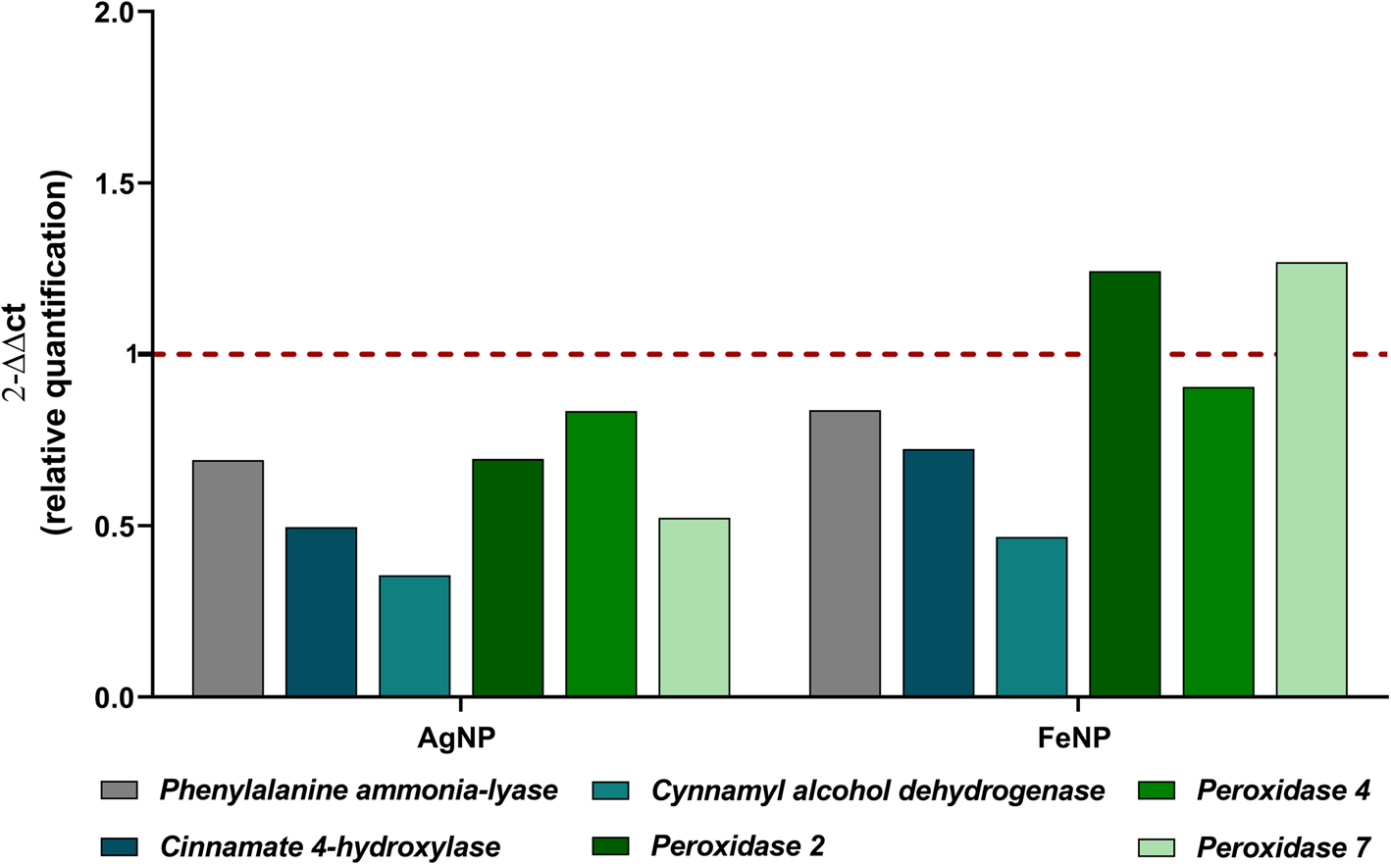
Effects of metallic nanoparticles in the expression of lignification-related genes that encode the enzymes phenylalanine ammonia lyase (*PAL*), cinnamate 4-hydroxylase (*C4H*), cinnamyl alcohol dehydrogenase (*CAD*), peroxidase 2 (*POD2*), peroxidase 4 (*POD4*) and peroxidase 7 (*POD7*) of soybean roots (*n* = 5). The red dotted line represents the quantification of negative control (soybean roots not exposed to the nanoparticles)

The increase in lignification is one of the defense responses of plants in stress conditions. When the plant is exposed to stress conditions the levels of H_2_O_2_ increase, triggering the up-regulation of the expression of lignification-related genes [35, 36]. In our study, AgNPs did not cause the up-regulation of lignification-related genes in the roots of soybean seedlings. However, FeNPs triggered the up-regulation of *POD2* and *POD7* genes (Fig. 5).

## Discussion

In the present study we evaluated the effects of biogenic metallic nanoparticles on morphological, physiological and biochemical parameters of soybean seedlings cultivated in soil exposed to these nanoparticles in greenhouse. The fact that most of the phytopathogens start their life cycle contaminating the plants via soil led us to perform the exposure through this route. In addition, planting in the soil and keeping the seedlings in a greenhouse allows better similarity with field cultivation conditions. Most studies found in the scientific literature investigated the phytotoxic effects of metallic nanoparticles in hydroponic systems or in culture media [37–39].

Analysis of the seedlings cultivated in the soil exposed to AgNPs demonstrated a low investment in root biomass and an increase in leaf area (Fig. 3), which could cause a water deficit due to lower soil water absorption and greater water loss by leaf surface transpiration, respectively [40]. However, a decrease in stomatal conductance without effects on the photosynthetic rate (Fig. 1) and photosynthetic leaf pigments (Fig. 2) was also observed. These changes may be considered an adaptive mechanism of the seedlings against the toxicity of AgNPs, since a higher intrinsic water use efficiency was observed.

It was reported that, in general, silver nanoparticles may cause negative effects on plants, such as reduction of chlorophyll levels, prejudices in nutrition, hormonal changes and alterations in transpiration and photosynthesis levels [41]. However, quite varied results can be expected when evaluating biogenic AgNPs. Negative morphological and physiological alterations such as the decrease of shoot and root length, decrease of biomass, and decrease of photosynthetic rates and photosynthetic pigments were observed in *Brassica* sp seedlings exposed to AgNPs synthesized using *Aloe vera* [42] and the bacteria *Bacillus marisflavi* [43], and also in *Lupinus termis* plants exposed to AgNPs based on the extract of *Coriandrum sativum* [44]. In contrast, some biogenic AgNPs have shown positive effects on the growth and physiology of plants. Biogenic AgNPs synthesized using leaves of *Eucaliptus globules* stimulated the germination and growth of *Zea mays* L., *Trigonella foenum-graecum* L., and *A. cepa* L. [45] and biogenic AgNPs synthesized using the fungi *Trichoderma harzianum* and *Aspergillus fumigatus* had stimulating effects on *Solanum lycopersicum* [21].

The biochemical analysis showed that the biogenic AgNPs caused an increase in the levels of H_2_O_2_ and conjugated dienes in the roots of soybean seedlings, indicating induction of oxidative stress (Fig. 4). The more pronounced effect on the roots in comparison with the leaves may be due to the direct contact of these structures with the AgNPs in the soil and may be linked to the lower dry weight in this part of the plant. Some studies reported the increase of biochemical markers of oxidative stress on plants exposed to biogenic AgNPs [42, 43, 46]. The analysis of lignification-related genes in the roots of soybean seedlings did not show up-regulation (Fig. 5). This result suggests that lignification was not employed by the seedlings as a defense mechanism against the exposure to the AgNPs [35, 47].

The analysis of the seedlings cultivated in the soil exposed to the FeNPs did not show many alterations compared to the control. In general, an increase in leaf area (Fig. 3) and an increase in H_2_O_2_ levels in the leaves were observed, without lipid peroxidation, not characterizing oxidative stress (Fig. 4). High levels of H_2_O_2_ in plants indicate the production of reactive oxygen species which may cause cell death, however low levels may induce defense mechanisms [31, 48]. The analysis of the expression of genes related to the lignification of the roots showed an increase in the expression of *POD2* and *POD7* genes (Fig. 5), indicating a defense response of the plants against the exposure to the FeNPs [47, 49], which may be consequently related to the absence of major alterations in most of the investigated parameters.

In general, iron nanoparticles are known to have lower toxicity and, in some cases, they are able to enhance plant development [50]. Specifically, few studies investigated the possible impacts of biogenic iron nanoparticles on plants, however most of them show favorable effects. Rajiv et al. (2017) evaluated the effects of iron oxide nanoparticles synthesized from the extract of *Lantana camera* plant against *Vigna mungo *in vitro and observed an increase in germination rate and shoot and root length [51]. Iron oxide nanoparticles synthesized from *Pistacia vera* also triggered beneficial effects on *Lycopersicon esculentum* seedlings increasing parameters such as seed vigor, shoot length and fresh and dry weight [52]. In an experiment with cell culture of *Cicer arietinum,* iron oxide nanoparticles synthesized with the extract of *Cymbopogon jwarancusa* promoted the increase of parameters related to plant growth such as callogenesis, regeneration dynamics and induction of shoot and root elongation [53]. Iannone et al. (2021) observed that magnetite nanoparticles coated with citric acid stimulated the growth of soybean and alfalfa plants with no effects on the levels of H_2_O_2_ and MDA [54]. In another study, iron oxide nanoparticles synthesized from the seaweed *Chaetomorpha antennina*, especially those coated with citrate, reduced drought stress on *Setaria italica* plants with the increase of biomass production and absence of toxic effects [55].

The need to investigate the possible effects of biogenic metallic nanoparticles on plants is emerging due to the potential that these new materials have been presenting for the control of phytopathogens which cause significant agricultural losses [10, 27, 56]. In addition, biogenic nanoparticles have a capping that confers unique characteristics, influencing their toxicity [9]. In a previous study by Guilger-Casagrande et al. (2021) it was found that the capping of silver nanoparticles synthesized from *T. harzianum* (the same employed in the present study) contributed to better stability and reduced toxic effects in most of the evaluations performed with cell cultures and non-target microrganisms. Evidences were also obtained that the synergy between nanoparticles and cappings enhance the antifungal effect of nanoparticles, however more studies are necessary [56].

Accordingly, it is essential to know the behavior of plants exposed to biogenic nanoparticles given their peculiarities associated with the different reducing and stabilizing agents employed in the synthesis. Further investigations may enable the exploration of the antimicrobial and phytostimulating properties of these nanoparticles in an environmentally friendly way, overcoming possible phytotoxic effects.

## Conclusion

This study demonstrated that the biogenic silver and iron nanoparticles had different effects on soybean seedlings, despite having the synthesis mediated by the same reducing and stabilizing agent. Some evidences of phytotoxicity followed by adaptive responses were observed in soybean seedlings grown in the soil exposed to AgNPs while stimulation of defense responses with no major effects were observed in those exposed to FeNPs. Given the well-known potential of biogenic metallic nanoparticles for the control of phytopathogens which affect agricultural crops, more investigations of the effects of these new nanomaterials on different plant species are necessary. In view of the above, perspectives are opened for the use of this type of nanomaterial as an effective alternative for solving agricultural problems in a safe and environmentally friendly way.

## Data Availability

The datasets generated during the current study are available in the Google Drive repository, [https://drive.google.com/drive/folders/1XmWjwgfT_q-zzYmSeEtJKA7p46GjC6sL?usp=sharing].
